# Performance of Xpert® MTB/RIF in diagnosing tuberculous pleuritis using thoracoscopic pleural biopsy

**DOI:** 10.1186/s12879-020-05578-3

**Published:** 2020-11-12

**Authors:** Chengjun Li, Chang Liu, Bingqi Sun, Wei Zhang, Yang Wang, Jiao Sun, Fang Ma, Yu Chen

**Affiliations:** 1 Department of Pleurisy, Shenyang Tenth People’s Hospital and Shenyang Chest Hospital, Shenyang, Liaoning Province 110044 People’s Republic of China; 2 Department of Thoracic Surgery, Shenyang Tenth People’s Hospital and Shenyang Chest Hospital, Shenyang, Liaoning Province 110044 People’s Republic of China; 3 Department of Tuberculosis Laboratory, Shenyang Tenth People’s Hospital and Shenyang Chest Hospital, Shenyang, Liaoning Province 110044 People’s Republic of China; 4 Department of Tuberculosis, Shenyang Tenth People’s Hospital and Shenyang Chest Hospital, Shenyang, Liaoning Province 110044 People’s Republic of China

**Keywords:** Tuberculous pleuritis, Xpert® MTB/RIF, Pleural biopsy, Diagnostics, Sensitivity, Specificity, Predictive value, MGIT 960

## Abstract

**Background:**

Etiological diagnosis of tuberculous pleuritis is challenging, owing to a paucity of *Mycobacterium tuberculosis* (MTB) in the affected region. Moreover, currently available methods, such as the detection of acid-fast bacilli and microbiological culture, are not always conducive to timely diagnosis and treatment. In this study, we evaluated the performance of Xpert® MTB/RIF assay (hereinafter referred to as “Xpert”) in detecting MTB in difficult-to-diagnose patients using suspensions of pleural biopsy tissue specimens obtained under direct thoracoscopic guidance.

**Methods:**

One hundred and sixty patients with an unexplained pleural effusion were included from the Shenyang Tenth People’s Hospital and Shenyang Chest Hospital, China, between 2017 and 2018. The included patients underwent thoracoscopy under local anesthesia, with an intercostal incision of approximately 1.0 cm for biopsy. The biopsy specimens were used for pathological and etiological examinations. The Xpert test was evaluated for its sensitivity and specificity, as well as positive and negative predictive values (PPV and NPV, respectively), against data obtained using standards: the BACTEC™ MGIT™ 960 liquid culture system and a composite reference standard (CRS).

**Results:**

The sensitivity and specificity of Xpert were 68.8 and 64.6%, respectively, against the MGIT 960 culture data. The PPV and NPV of Xpert were 56.4 and 75.6%, respectively. The sensitivity of Xpert was 69.0% against the CRS data, which was significantly higher than that of MGIT 960 culture (56.6%). The PPV and NPV of Xpert against the CRS data were 100.0 and 57.3%, respectively.

**Conclusions:**

Xpert is a good rule-in test but has limited value as a rule-out test for the diagnosis of tuberculosis pleuritis.

## Background

Tuberculosis (TB) is one of the top 10 causes of death worldwide and the leading cause of death from a single infectious agent (ranking higher than HIV/AIDS), according to the Global Tuberculosis Report 2019 released by the World Health Organization (WHO) [1]. According to the 2017 estimates, 27% of the global incidence occurred in India, 9% in China, and 8% in Indonesia; thus, these top three countries account for 44% of the global incidence [2]. In 2016 and 2017, 836,236 and 835,193 cases of pulmonary TB were reported in China, respectively [3, 4]. Although the majority of TB cases are pulmonary, approximately 25% of adult TB cases are extrapulmonary, most of which involve lymph nodes and the pleura as the initial affected sites [5].

Tuberculous pleuritis is the main cause of a pleural effusion in some countries [6]. The current gold standard for the diagnosis of tuberculous pleuritis is the drug sensitivity test using the BACTEC MGIT 960 liquid culture system. However, this diagnostic method has a low positive rate and is laborious and time consuming, which often delays the diagnosis and treatment.

Currently, a molecular detection method, the Xpert® MTB/RIF assay (hereinafter referred to as “Xpert”; Cepheid, Sunnyvale, CA, USA), which is an automated, cartridge-based nucleic acid amplification test for *Mycobacterium tuberculosis* (MTB), is being used increasingly. This method can simultaneously detect the MTB nucleic acid and resistance to rifampicin (RIF) [7]. Therefore, the WHO recommended this method for the diagnosis of TB in 2010 [8]. In 2013, the WHO expanded its application for the diagnosis of extrapulmonary TB in adults and children, although the samples tested were limited to cerebrospinal fluid, lymph node fluid, and tissue specimens [9]. The pooled sensitivity and specificity of Xpert in the diagnosis of tuberculous pleuritis using tuberculous pleural effusion (TPE) fluid were shown to be 51.4 and 98.6%, respectively [10–12]. The low sensitivity and a limited diagnostic capacity of Xpert are probably due to a low organism burden in the pleural fluid [13]. Studies have shown that pleural biopsies, obtained under medical thoracoscopy guidance, are better specimens than the pleural fluid for diagnosing tuberculous pleuritis [9, 14, 15]. Our group has previously evaluated the performance of Xpert using urine specimens for the rapid diagnosis of urinary tract TB, and the data indicated a better performance of Xpert than that of smear and culture identification, thus suggesting that Xpert could be considered for the diagnosis of urinary tract TB [16].

The BACTEC™ MGIT™ 960 liquid culture system (Becton Dickinson) and or clinical composite reference standards (CRSs) are used as the gold standards for TB diagnosis. CRSs are often used as a standard for comparison of test performance in detecting extrapulmonary TB, as culture is suboptimal in this case [17, 18]. In the present study, our primary objective was to evaluate the performance of Xpert against that of MGIT 960 culture and a CRS in detecting MTB in difficult-to-diagnose cases, using suspensions of pleural biopsy tissue specimens for the rapid diagnosis of tuberculous pleuritis. Our secondary objective was to evaluate the performance of Xpert in detecting RIF-resistant MTB against that of the gold standard, namely, the MGIT 960 culture system.

## Methods

### Subject recruitment

We screened all suspected patients with a pleural effusion, who visited the Shenyang Tenth People’s Hospital and Shenyang Chest Hospital between January 1, 2017, and December 31, 2018, using established procedures. After excluding patients in whom tuberculous pleuritis was confirmed by clinical or microbiological analysis of the pleural fluid, undetermined cases were invited to undergo thoracoscopy. The enrollment criteria were as follows: color ultrasound examination showing a unilateral or bilateral pleural effusion; the effusion depth > 3 cm; no prior anti-TB treatment or thoracentesis; a general status that would allow tolerating the internal thoracoscopic operation; and a negative HIV test.

### Tuberculous pleurisy and malignant tumor diagnoses

The etiological diagnosis included the identification of MTB in pleural biopsy specimens by smear or culture [18]. The histopathological diagnosis included the identification of caseous granuloma via histopathological testing of thoracic tissue specimens and the identification of pleurisy via acid-fast staining positivity [19]. The diagnosis of a malignant pleural effusion was based on the identification of clear malignant cells via pathological examination of pleural tissue [20].

### Thoracoscopy procedure

All patients included in this study had diagnostic indications for thoracoscopy but not for needle biopsy; thus, they did not undergo needle biopsy. Only a small amount of tissue with diagnostic significance was produced by needle biopsy and it often yielded negative results. Therefore, it was impossible to evaluate the pleural condition by needle biopsy. The thoracoscopy procedure is summarized as follows: color ultrasound positioning was performed to determine the surgical incision site. After administering a local anesthetic, an intercostal incision of approximately 1.0 cm in length was made, and the subcutaneous tissue was separated until the pleural cavity was reached. A flexible electronic thoracoscope (model LTF-240; Olympus, Japan) was pushed along a cannula into the chest cavity to scan the pleural cavity, and manually controlled negative pressure was used to aspirate the pleural fluid; the visceral and parietal surfaces were observed and imaged. Lesions in the pleura were then biopsied to assist the diagnosis.

### Sample preparation and diagnostic tests

#### Specimen processing

The pleural biopsy specimens were divided into two parts. The one for pathological examination was fixed with 4% formaldehyde, embedded in paraffin, and sliced for acid-fast staining and hematoxylin/eosin staining. The other part was used for the detection of MTB, for which a minced sample was transferred to a 2-mL sample tube containing sterile ceramic beads with a diameter of 6 mm, and 0.5 to 1.0 mL of 0.9% sodium chloride for injection was added. The lid was tightened, and the sample tube was inserted into a FastPrep®-24 homogenizer (MP Biomedicals, USA), with a frequency set to 6.5 m/s, time to 20 s, and general tissue oscillation to two to three times. The prepared suspension was then used for MGIT 960 culture and the Xpert test. The main culture and test steps were as follows: 2 mL of a suspension sample and 1–2 volumes of 2% *n*-acetyl-L-cysteine NaOH sodium citrate were vortex shaken for 20 s, followed by incubation at room temperature for 15 min. The volume was adjusted to 45 mL with phosphate-buffered saline (PBS; pH 6.8), and the resulting mixture was centrifuged at 3000×*g* at 4 °C for 15 min. After removal of the supernatant, the pellet was resuspended in 1.5 mL of PBS.

#### MGIT 960 culture

A 0.5-mL volume of the resulting suspension was placed in an MGIT 960 liquid culture tube and incubated at 37 °C for 42 days. After the instrument automatically reported a positive culture on day 42, the culture was smeared for microscopy, and 0.1 mL of the Auramine O dye was added for Ziehl–Neelsen staining, followed by fluorescence microscopy. If acid-fast bacilli (AFB) were detected, the result was considered true positive. If no AFB were found, the result was read as negative. All steps followed the guidelines of the MGIT 960 system manual [21]. Positive MTB cultures were then tested for susceptibility to RIF (40 μg/mL). For positive isolates, drug sensitivity tests for isoniazid, streptomycin, and ethambutol were also performed.

#### Xpert test

For an Xpert test [9], 1 mL of a sample was processed as described above, with 2 mL of the sample buffer added, followed by incubation on a vortex oscillator for 15 s. Next, 2 mL of the liquefied sample was pipetted into an Xpert cartridge and placed into an Xpert detection system module (Cepheid, USA) to start automated testing. After approximately 2 h of reaction, the test results of MTB detection and RIF resistance could be directly observed on the detection system window.

### Statistical and data analysis

Stata 14.1 (Stata Corp, College Station, TX, USA) was used for statistical analysis. A chi-squared test was used for categorical variables, and a Student’s *t*-test was used for continuous normally distributed variables. For continuous not normally distributed variables, the Wilcoxon rank-sum test was used. The chi-squared test was also utilized for statistical comparison of the sensitivity of different methods. A *P*-value of < 0.05 was considered statistically significant.

The performance of Xpert was first compared with that of MGIT 960 culture. Afterward, using the CRS or the final diagnosis as the gold standard, the performances of Xpert and MGIT960 were evaluated, including the sensitivity, specificity, positive predictive value (PPV), and negative predictive value (NPV). The CRS used in this study referred to the final diagnosis based on one of the following criteria: 1) positive MGIT 960 culture; 2) histopathological manifestations; and 3) thoracoscopic signs and improvement after anti-TB treatment [19].

### Ethics statement

The protocol of the study was reviewed and approved by the Ethical Committee of the Shenyang Chest Hospital. Complete clinical data were recorded for all patients, who provided informed consent prior to examination.

## Results

### Participants

**Fig. 1:**
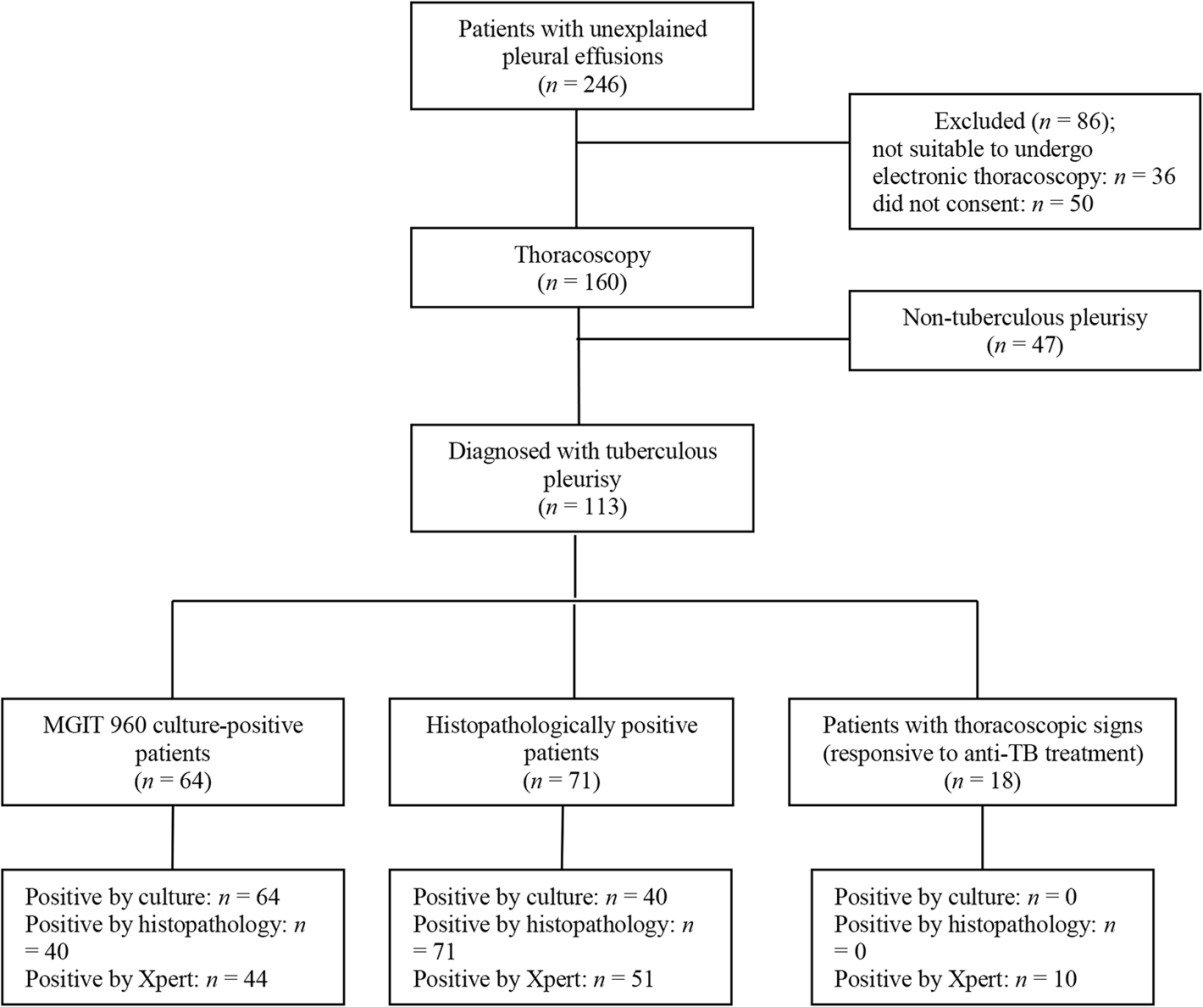
Flow diagram of patient enrollment, allocation, and analysis. TB: tuberculosis

A total of 523 patients with a pleural effusion visited the Shenyang Tenth People’s Hospital and Shenyang Chest Hospital between January 1, 2017, and December 31, 2018. Among these, 251 were confirmed to have pleural TB, and 26 were confirmed to have a malignancy. Among the former 251 patients, the diagnosis of pleural TB was made via laboratory examination of pleural effusions in 56 patients (3 by smear microscopy, 25 by MGIT 960 culture, and 28 by Xpert) and via clinical diagnosis in 195 patients. Of the remaining 246 patients with an unexplained pleural effusion, 36 were not suitable to undergo electronic thoracoscopy. Another 50 patients refused the electronic thoracoscopic procedure. Finally, 160 patients with an unexplained pleural effusion were enrolled in this study and underwent thoracoscopy. The detailed flow diagram of the study is presented in Fig. 1.

**Fig. 2:**
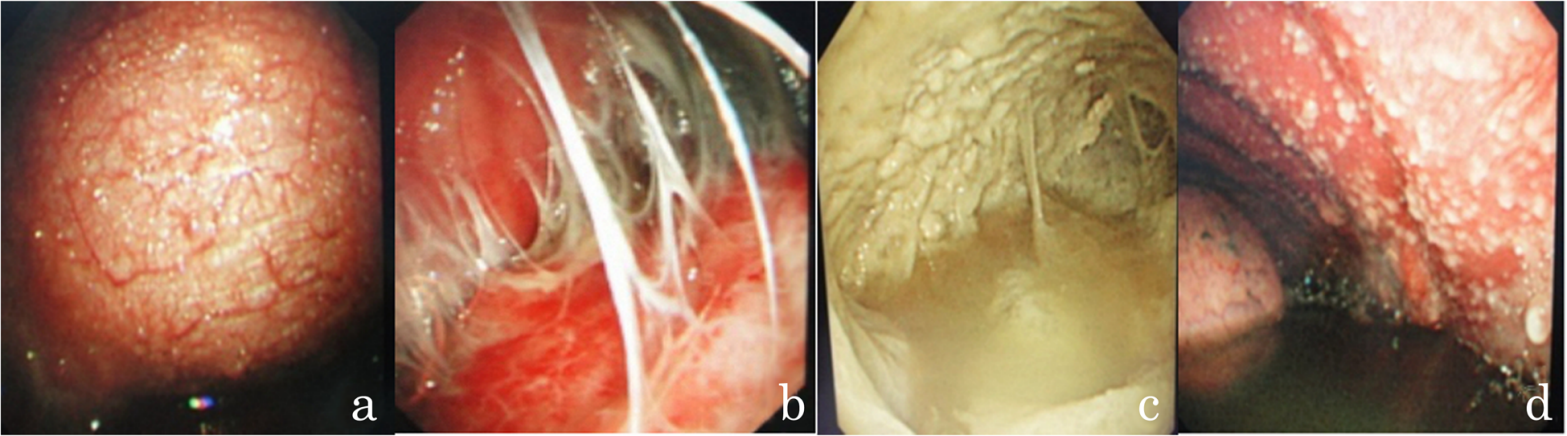
Thoracoscopic tuberculous pleurisy. **a** Hyperemic, edematous, and thickened pleura. **b** Pleural adhesions or fibrotic septa. **c** Necrosis scattered over the pleura. **d** Diffuse miliary nodules (< 5 mm) on the pleura

**Fig. 3:**
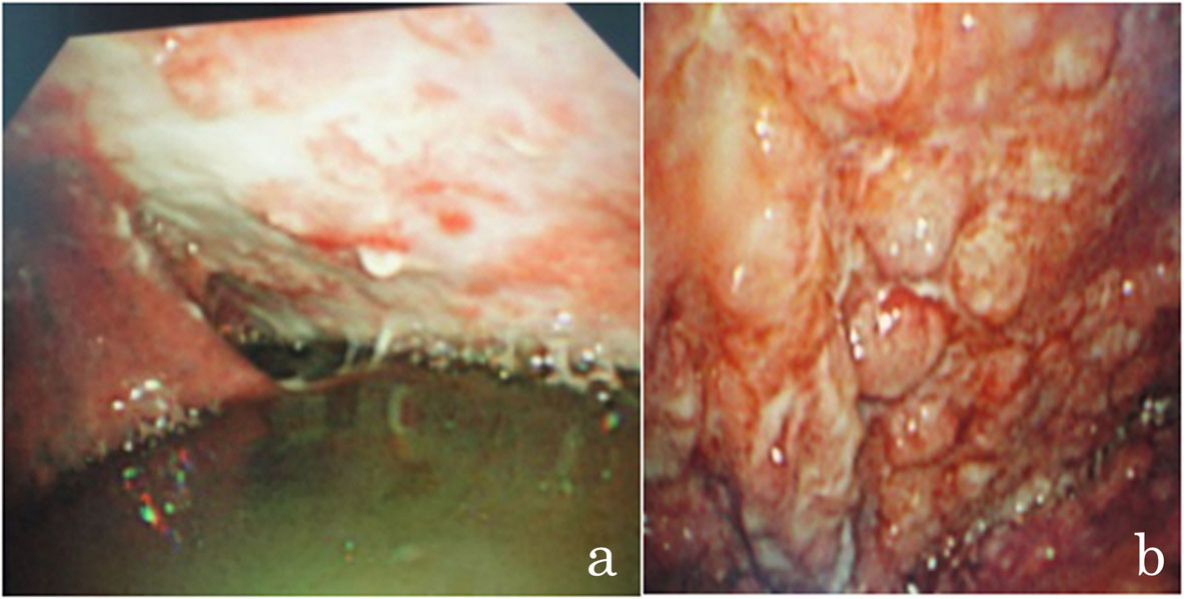
Thoracoscopic malignant pleural metastasis. **a** Metastatic pleural tumor (squamous cell carcinoma). **b** Pleural mesothelioma

Among the 160 patients, 98 (61.2%) were males, and 62 (38.8%) were females. The ages of the participants ranged from 14 to 77 years, with an average of 49.2 ± 15.4 years. According to the CRS, 113 (70.6%) patients were diagnosed with tuberculous pleurisy; 64 were positive by MGIT 960 culture; 71 were positive by histopathology (40 were positive by both MGIT 960 culture and histopathology); and 18 had thoracoscopic signs and were responsive to anti-TB treatment. Thoracoscopy showed different morphologies of TPEs, such as hyperemic, edematous, and thickened pleura; pleural adhesions or fibrotic septa; necrosis scattered over the pleura; and diffuse miliary nodules (< 5 mm) on the pleura (Fig. 2). There were 47 (29.4%) patients with non-tuberculous pleurisy. Of these, 31 (66.0%) patients had a malignancy, 6 (12.8%) had a suspected malignancy, and 10 (21.3%) had other conditions (Table 1 and Fig. 3).

**Table 1:** Comparison of participants with and without tuberculous pleuritis

Characteristics	Overall (*n* = 160)	Pleural tuberculosis (*n* = 113)	No pleural tuberculosis (*n* = 47)	*P*-value
Age (years)	49.2 ± 15.4	45.9 ± 15.7	57.2 ± 11.2	< 0.001
Males, *n* (%)	98 (61.2)	74 (65.5)	24 (51.1)	0.088
Disease duration (weeks)	6.5 ± 8.6	4.9 ± 5.9	10.5 ± 12.1	< 0.001

Age and duration are expressed as the mean ± standard deviation

### Performance of Xpert in diagnosing tuberculous pleuritis using thoracoscopic pleural biopsy specimens

The performance of Xpert was evaluated using MGIT 960 liquid culture as the gold standard. The sensitivity of Xpert was 68.8% [44/64; 95% confidence interval (CI): 55.8–79.4%], and the specificity was 64.6% (62/96; 95% CI: 54.1–73.9%). Under the same standard, the PPV of Xpert was 56.4% (44/78; 95% CI: 44.7–67.4%), and the NPV was 75.6% (62/82; 95% CI: 64.7–84.1%).

Compared with the CRS, the sensitivity of Xpert was 69.0% (78/113; 95% CI: 59.5–77.2%), and the specificity was 100.0% (95% CI: 90.6–100%). Using the same standard, MGIT 960 culture showed a sensitivity of 56.6% (64/113; 95% CI: 47.0–65.8%), which was significantly lower than that of Xpert (*P* <  0.05), while the specificity was also 100.0%. Using the CRS as a reference, the PPVs of Xpert and MGIT 960 culture were both 100.0%. The NPV of Xpert was 57.3% (47/82; 95% CI: 45.9–68.0%), which was higher than that of MGIT 960 culture at 49.0% (47/96; 95% CI: 38.7–59.3%), although the difference was not statistically significant (Table 2).

**Table 2:** Sensitivity, specificity, and positive and negative predictive values of Xpert MTB/RIF and MGIT 960 culture in detecting tuberculous pleuritis

Test	Sensitivity, % (95% CI)	Specificity, % (95% CI)	Positive predictive value, % (95% CI)	Negative predictive value, % (95% CI)
MGIT 960 culture (*n* = 64)				
Xpert MTB/RIF	68.8 (55.8–79.4)	64.6 (54.1–73.9)	56.4 (44.7–67.4)	75.6 (64.7–84.1)
CRS (*n* = 113)				
Xpert MTB/RIF	69.0 (59.5–77.2)	100.0 (90.6–100.0)	100.0 (94.2–100.0)	57.3 (45.9–68.0)
MGIT 960 culture	56.6 (47.0–65.8)	100.0 (90.6–100.0)	100.0 (92.9–100.0)	49.0 (38.7–59.3)

*CRS* composite reference standard, *CI* confidence interval

### Detection of antibiotic resistance

Among all participants, 13 patients were found to be infected with MTB resistant to one or more antibiotics, including 3, 6, 2, and 2 isolates resistant to streptomycin only, RIF only, streptomycin and isoniazid, and RIF and isoniazid, respectively. MGIT 960 culture identified resistant isolates in 12 out of the 13 patients, missing only 1 isolate with RIF resistance. Xpert identified all isolates resistant to RIF.

## Discussion

In the present study, we evaluated the performance of Xpert in detecting MTB in patients with unexplained pleural effusions using suspensions of pleural biopsy tissue specimens collected during limited thoracic surgery. The results were compared with those obtained using the MGIT 960 mycobacterial culture system and a CRS. The present study followed an orderly sequence of diagnostic steps, namely, testing pleural effusions and using other clinical diagnostic methods. Patients with unexplained pleural effusions were invited to join this study and undergo thoracoscopy if they met the inclusion criteria.

Previous studies have reported that the pooled sensitivity of Xpert in diagnosing tuberculous pleuritis using TPE fluid was between 30 and 51% [10–12]. A previous study that employed Xpert to diagnose tuberculous pleuritis using pleural biopsy specimens revealed that the pooled sensitivity and specificity were 30.5% (95% CI: 3.5–77.8%) and 97.4% (95% CI: 92.1–99.3%), respectively, using culture results as the reference standard, although one study reported the sensitivity to be as high as 85.5% [22]. Another study evaluated the performance of Xpert and reported that the sensitivity was 16.0% (95% CI: 5.0–36.0%) and 13.3% (95% CI: 4.0–31.0%) against that of two CRSs. Thus, the authors suggested that Xpert is not the ideal diagnostic tool for pleural TB [23]. Subsequently, the same team improved their methods, thereby increasing the sensitivity of Xpert in diagnosing tuberculous pleuritis to 45.0% (95% CI: 32.1–58.4%) [15]. The authors explained that the low sensitivity in their previous study was probably due to a small amount of pleural tissue obtained using a closed pleural biopsy.

In the present study, the sensitivity of Xpert was 68.8 and 69.0% for the diagnosis of tuberculous pleuritis, respectively against the MGIT 960 and CRS data, which demonstrated a great potential of Xpert as a diagnostic tool for suspected difficult-to-diagnose cases of tuberculous pleuritis using thoracoscopic pleural biopsy specimens. The Xpert method yields results in only 2 h, whereas MGIT 960 culture requires at least 2 to 3 weeks. The Xpert test thus offers a considerable advantage over the MGIT 960 culture method in terms of the time required for diagnosis. The latest diagnostic criteria for pulmonary TB in China include positive detection of the MTB nucleic acid in the pleural fluid for the diagnosis of tuberculous pleurisy [24].

Tuberculous pleurisy is a serious disease caused by MTB infection. The gold standards for the diagnosis of tuberculous pleuritis include microbiological tests, such as smear testing for AFB and culture to detect MTB in pleural effusions [25]. However, pathogen detection is extremely difficult, owing to a limited number of bacteria in a pleural effusion. Thus, the sensitivity could have been overestimated in several previous studies that used AFB and culture as the gold standards. Moreover, late and missed diagnoses and treatment have great impacts on patients’ prognosis.

Although internal thoracoscopy is invasive, it is usually an easy procedure that involves a small incision, and can be performed under local anesthesia, with a clear vision, safety, and efficacy [26]. The diagnostic efficiency of thoracoscopy for tuberculous pleuritis has been reported to reach as much as 93.4% via performing biopsy under direct visual control [27]. Another study reported a diagnostic efficiency of 92.6% in 833 patients with an unexplained pleural effusion, who were diagnosed using thoracoscopic medical examination [28]. A meta-analysis including 17 studies and 755 patients with an unexplained exudative pleural effusion revealed a pooled sensitivity of 91.0% [29]. Therefore, medical thoracoscopy can be used effectively for the diagnosis of unexplained pleural effusions.

Based on the results of the present study, pleural biopsy tissue suspensions can also be used effectively in the future for the diagnosis of tuberculous pleuritis using Xpert and culture for confirmed positive results. As the number of patients infected with antibiotic-resistant MTB was insufficient, it was difficult to draw a conclusion regarding the performance of Xpert in the diagnosis of resistant TB.

## Conclusions

The use of internal thoracoscopic biopsy tissue suspensions for MTB detection using Xpert demonstrated the feasibility and sensitivity of the test, which can be applied in clinical settings as a diagnostic tool for tuberculous pleurisy. The test offered the following advantages: (1) collecting pleural biopsy specimens during thoracic surgery improved the diagnostic accuracy; (2) grinding the specimens allowed their use for different tests, including MGIT 960 culture and Xpert. One of the key limitations is that the preprocessing procedure was too harsh and may have reduced the bacterial load for culture; yet, this was not a problem for Xpert; (3) the positive rate of detection using Xpert with biopsy tissue was significantly higher than that obtained with pleural fluid, which is more meaningful for clinical diagnosis. However, the results may have been affected by the small sample size and limited specimen volume obtained using pleural biopsy. In the future, more clinical data are needed to confirm the value of Xpert MTB/RIF in diagnosing tuberculous pleuritis.

## Data Availability

Data generated or analyzed during this study are included in this manuscript; the remaining data are available from the corresponding author on reasonable request.

## Abbreviations

AFB: Acid-fast bacilli
CI: Confidence interval
CRS: Composite reference standard
MTB: *Mycobacterium tuberculosis*
NPV: Negative predictive value
PBS: Phosphate-buffered saline
PPV: Positive predictive value
RIF: Rifampicin
TB: Tuberculosis
TPE: Tuberculous pleural effusion
WHO: World Health Organization
